# A Gas Chromatography-Mass Spectrometry Based Study on Urine Metabolomics in Rats Chronically Poisoned with Hydrogen Sulfide

**DOI:** 10.1155/2015/295241

**Published:** 2015-04-14

**Authors:** Mingjie Deng, Meiling Zhang, Fa Sun, Jianshe Ma, Lufeng Hu, Xuezhi Yang, Guanyang Lin, Xianqin Wang

**Affiliations:** ^1^Analytical and Testing Center of Wenzhou Medical University, University-Town, Wenzhou 325035, China; ^2^The First Affiliated Hospital of Wenzhou Medical University, Wenzhou 325000, China

## Abstract

Gas chromatography-mass spectrometry (GS-MS) in combination with multivariate statistical analysis was applied to explore the metabolic variability in urine of chronically hydrogen sulfide- (H_2_S-) poisoned rats relative to control ones. The changes in endogenous metabolites were studied by partial least squares-discriminate analysis (PLS-DA) and independent-samples *t*-test. The metabolic patterns of H_2_S-poisoned group are separated from the control, suggesting that the metabolic profiles of H_2_S-poisoned rats were markedly different from the controls. Moreover, compared to the control group, the level of alanine, d-ribose, tetradecanoic acid, L-aspartic acid, pentanedioic acid, cholesterol, acetate, and oleic acid in rat urine of the poisoning group decreased, while the level of glycine, d-mannose, arabinofuranose, and propanoic acid increased. These metabolites are related to amino acid metabolism as well as energy and lipid metabolism *in vivo*. Studying metabolomics using GC-MS allows for a comprehensive overview of the metabolism of the living body. This technique can be employed to decipher the mechanism of chronic H_2_S poisoning, thus promoting the use of metabolomics in clinical toxicology.

## 1. Introduction

Hydrogen sulfide (H_2_S), a lethal gas best known for colorlessness, irritant, asphyxiant, and smelling like rotten eggs [1–4]. It generally occurs as an environmental contaminant in unrefined natural gas and petroleum, sulfur deposits, volcanic gases, well water, sulfur springs, or many other industrial manufacturers [2]. The toxicological versus biological profiles of H_2_S as well as the chemical properties of H_2_S have been well characterized [5, 6]. Hundreds of years of human knowledge on H_2_S are nothing compared with H_2_S-caused lethal and sublethal effects. Growing evidence has shown that H_2_S is a gaseous messenger produced in mammalian cells and turns out to play key roles in the body [4, 5, 7].

H_2_S causes systemic toxic lesions. Exposure to H_2_S can occur by inhaling the substance, by eating or drinking it, or by skin contact. Ingestion of H_2_S has been reported to cause bronchiolitis, pulmonary edema, reactive airway disease, pulmonary interstitial fibrosis, and death [8]. The toxic effects are due to inhibiting the mitochondrial respiratory by the hydrogen sulfide ion HS^−^ operating on cytochrome oxidase, selectively on cytochrome-C oxidase [9, 10]. The degree of poisoning depends on the length of exposure and concentration of H_2_S [11]. A higher concentration of H_2_S inhibits the central nervous system, while a lower concentration of H_2_S is a mucosal irritant. Long-term contact with small quantities of H_2_S also shows teratogenic effects and is one of the important predisposing factors in occupational tumors.

Fatal poisonings are classically described in an occupational environment, especially in sewer workers and employees in the industries mentioned earlier [10]. Poisonings were very recently reported in an agricultural environment, during the fermentation of cereals, liquid manure, or slurry [12]. Furthermore, suicides and accidental poisonings in a domestic setting are still exceptional [10, 13]. Therefore, for the diagnosis of H_2_S poisoning, multivariate statistical analysis of the major metabolite of H_2_S, in biological fluids, such as blood and urine, is necessary.

Metabolomics is defined as “the quantitative measurement of the dynamic multiparametric metabolic response of living systems to pathophysiological stimuli or genetic modification” [14, 15]. It can harmlessly and dynamically detect changes in metabolites in biological fluids, evaluate the toxic effect of the substances being tested, provide information related to drug toxicity, and evaluate the clinical effects of drugs on gene expression by integrating its temporal and spatial effects [16, 17]. GC-MS based metabolomics combines gas chromatography-mass (GC-MS) spectroscopic profiling of biological samples with multivariate statistical analysis [18–20], as such GC-MS based metabolomics is well established as a powerful technique for rapidly identifying changes in the global metabolite profiles of biological samples since GC-MS had a more complete database of mass spectra [21, 22].

In previous study, it should pay close attention to drug interactions when take drugs metabolized through CYP1A2 and CYP2B6 enzyme after hronic hydrogen sulfide poisoning [23]. Another critical issue that makes the biological role of H_2_S controversial is the lack of general consensus about subtle metabolic perturbation of H_2_S poisonings in urine and plasma. Metabolic data derived from urine samples can provide a systemic approach to investigating the detailed metabolic mechanisms of H_2_S poisoning. We performed urinary GC-MS based metabolomics analysis obtained from H_2_S-poisoned rats, as well as their age-matched controls. The purpose of this analysis was to extend the scope of our previous studies and further understand the pathogenesis of H_2_S poisoning on systemic and metabolic levels. The results of our study can facilitate the understanding of the complicated, potential molecular mechanisms of H_2_S poisoning and its complications.

## 2. Experiment

### 2.1. Instruments and Reagents

6890N/5975C gas chromatography-mass spectrometer (America Agilent), Agilent 7683 automatic sampler, Agilent HP-5MS capillary column (30 m ∗ 0.25 mm ID ∗ 0.25 *μ*m), Agilent chromatographic work station, and NIST 2008 mass spectrometry database were applied as instruments and data foundation in the experiment. Methoxylamine hydrochloride (analytically pure, Sigma), N-methyl-N-methyl-trimethyl-silyl-trifluoroacetamide-trimethylchlorosilane (MSTFA-TMCS) (analytically pure, Sigma), pyridine (analytically pure, Fluka), acetonitrile (chromatographically pure, Sigma), n-heptane (analytically pure, Aladdin Reagent Co., Ltd., Shanghai), and helium (He) (high-grade pure, Shanghai BOC Gas Industry Co. Ltd.) were adopted as experimental reagents.

### 2.2. Animal Model of H_2_S Poisoning

Forty-nine male Sprague-Dawley rats with body weight of 250 ± 10 g were purchased from Shanghai Silaike Experimental Animal Co., Ltd., with an animal permit number of SCXK (Shanghai) 2012-0005. The rats were fed food and water ad lib and housed at 22°C. All operational procedures and protocols of the animal experiment were implemented strictly following the* Experimental Animals Use and Protection Guidelines* issued by the Animal Protection and Use Committee of Wenzhou Medical University. The rats were placed in a toxicant exposure cabinet equipped with an internal H_2_S detector. Some rats (*n* = 35) were randomly selected and exposed to 20 ppm H_2_S gas over a period of 40 days (twice a day in the morning and evening, 1 h per exposure) to prepare chronic H_2_S poisoning model. And others (*n* = 14) were exposed to the same volume of air with 0 ppm H_2_S gas as the control.

Each urine sample was collected for 24 h after 12 h fasting at the time points of 40 days after H_2_S gas exposing, respectively. The urine samples were collected over ice into 0.1 mL of 1% sodium azide solution and then centrifuged for 10 min at 4°C. The supernatant was stored at −80°C until measurement.

### 2.3. Sample Pretreatment and GC-MS Analysis

After thawing, 100 *μ*L of urine was added to 250 *μ*L of acetonitrile and was placed on an ice bath for 10 min after mixing thoroughly. The solution was centrifuged at 10,000 rpm for 10 min. The solution (250 *μ*L) was drawn from the top, placed in the reaction bottle, and dried using N2. Fifty *μ*L of 15 mg/mL pyridine methoxyl amine solution was added to the reaction bottle and thoroughly mixed. The solution was oximated at 70°C for 1 h and 50 *μ*L derivatization reagent (MSTFA : TMCS = 100 : 1, V/V) was added. The combined solution was left to incubate for 1.0 h following mixture. After incubation, 100 *μ*L n-heptane was added, sufficiently mixed, and centrifuged to separation (3,000 r/min for 10 min). The liquid supernatant was drawn and placed in the sample injection tube for GC-MS analysis.

GC-MS analysis conditions: sample injection is under a temperature of 270°C, without split sampling; the sample volume is 1 *μ*L and the solvent delay continues for 5 min; as for the temperature programming, its initial temperature is 85°C, retained for 5 min; then the temperature rises from 85°C to 300°C at a speed of 10°C/min, retained for 5 min; the interface temperature is set at 280°C and ion source temperature is 230°C; the ionization voltage stays at −70 eV; the quadrupole temperature is 150°C; helium (He) serves as the carrier gas, with a flow rate of 1.0 mL/min; the full scan is conducted at 50 to 600 m/z.

### 2.4. Data Processing

The Agilent chromatography work station was used to integrate the peak areas corresponding to various metabolites in the total ion chromatography using GC-MS, and the data was exported to Microsoft Excel, with the peaks normalized to the total sum of spectrum prior to multivariate analyses. The concentrations of the metabolites were expressed as relative peak areas. After removal of overloaded metabolite peaks in GC-MS analysis, the metabolite data derived from the control and chronic poisoning groups were imported into SIMCA-P 12.0 software (Umetrics, Umea, Sweden) for partial least squares-discriminate analysis (PLS-DA) processing. Independent-samples* t*-test was used (SPSS16.0 software, IBM) to analyze difference between metabolite groups, and a *P* value of <0.05 was considered statistically significant. The endogenous metabolites in the urine were identified using the NIST 2008 mass spectrometry database.

## 3. Results and Discussion

### 3.1. GC-MS Metabolite Spectrum Analysis

**Figure 1:**
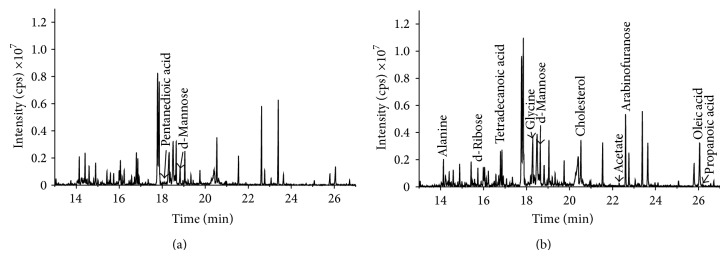
GC-MS spectra of urinary samples obtained from one control rat (a) and one model rat (b), respectively.

The GC-MS total ion chromatography (TIC) of rat urine is shown in Figure 1. By derivatizing semivolatile and nonvolatile metabolites, the levels of various metabolites in biological fluids were determined by GC-MS. More than 100 metabolites were detected in the urine, 17 of which are listed in Table 1 (with a degree of matching above 80%). The experiment results show that GC-MS analysis has high stability and reproducibility and the RSD (relative standard deviation) of each common peak was less than 15% (*n* = 6). The urea in the urine was overloaded on account of its high concentration and thus interfered with compounds with a retention time of less than 12 min. Therefore, only compounds with a retention time longer than 12 min were investigated. GC-MS analysis suggested that the metabolite spectrum in the urine mainly included contributions from amino acids, organic acids, carbohydrates, and lipids. The concentrations of these substances inside body are related to the metabolism of amino acid, energy, and lipids inside the organism.

### 3.2. Difference in Metabolite Spectra between Control and H_2_S-Poisoned Rats

**Figure 2:**
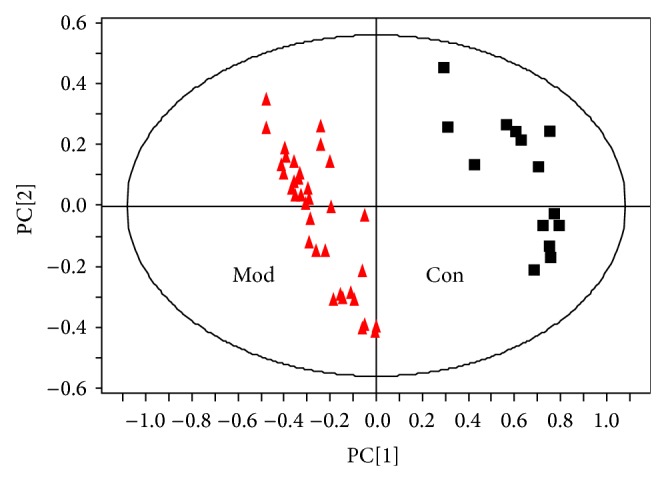
PLS-DA score plots based on GC-MS spectra of urine samples from rats of chronical hydrogen sulfide poisoning group (Mod) and control group (Con), (▲ model group, ■ control group).

Representative GC-MS spectra of urine samples obtained from rats in the control and H_2_S-poisoned group are shown in Figure 1, respectively. Using the NIST 2008 mass spectrum database, 17 endogenous metabolite peaks were identified and compared to the control group; the level of alanine, d-ribose, tetradecanoic acid, L-aspartic acid, pentanedioic acid, cholesterol, acetate, and oleic acid in the urine of H_2_S-poisoned rats reduced (*P* < 0.05), while the level of glycine, d-mannose, arabinofuranose, and propanoic acid increased (*P* < 0.05). PLS-DA method (SIMCA-P 12.0 software) was used to further study the metabolite spectra differences between control rats and H_2_S-poisoned rats. As shown in Figure 2, the sample points of the control group and the H_2_S poisoning model group are completely separated, suggesting that the overall metabolism of the H_2_S-poisoned rats changed significantly. The outliers, respectively, in the control group and the H_2_S poisoning group were probably caused by individual difference.

### 3.3. Metabolomics Study

Metabolomics is quickly becoming one of the indispensable methods for the study of systems biology, following genomics, transcriptomics, and proteomics. Metabolomics studies the downstream products of genes and proteins and reflects the end product of biological regulatory mechanisms. Thus, data obtained from metabolomics relates more closely to the final phenotype than data from genomics and proteomics. Subtle changes at the genetic and protein levels can be magnified in the metabolites, thereby facilitating detection of the final regulatory outcome. As a result, metabolomics are becoming more widely applied to study the pathogenesis, diagnosis, and prognosis of diseases.

**Figure 3:**
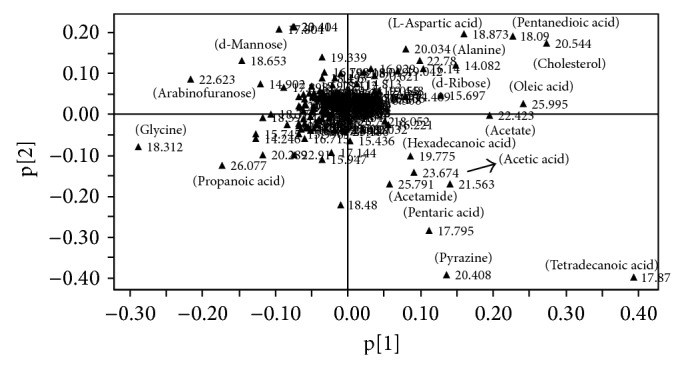
PLS-DA loading plot revealing the metabolites with large intensities responsible for the discrimination of the corresponding score plots (Figure 2).

In this study, the results showed that the first principal components of the rats in the chronical H_2_S poisoning group were distinguished from the rats in the control group (Figure 2). The corresponding load diagram (Figure 3) showed marked changes in the levels of key endogenous metabolites that separated H_2_S poisoning group from control group were alanine, d-ribose, tetradecanoic acid, L-aspartic acid, pentanedioic acid, cholesterol, acetate, oleic acid, glycine, d-mannose, arabinofuranose, and propanoic acid, This result can provide in detail the mechanism of chronical H_2_S poisoning and help in the early diagnosis and prevention of H_2_S poisoning and its complications.

### 3.4. Changes in Metabolite

The variations of metabolites allowed us to receive some important metabolic information about the mechanisms involved in H_2_S poisoning. Actually, in our work, systemic abnormalities happening in H_2_S-poisoned rats can lead to pathological changes of several metabolites in plasma and, consequently, to the urinary overflow [24].

Alanine is a nonessential amino acid made in the body from the conversion of the carbohydrate pyruvate or the breakdown of DNA and the dipeptides carnosine and anserine. It is highly concentrated in muscle and is one of the most important amino acids released by muscle, functioning as a major energy source [25, 26]. D-ribose is commonly referred to simply as ribose, a five-carbon sugar found in all living cells. Ribose is a structural component of ATP, which is the primary energy source for exercising muscle [27]. Aspartic acid is one of the 20 natural proteinogenic amino acids which are the building blocks of proteins, and it played important roles in the urea cycle and DNA metabolism [28]. Cholesterol is essential for all animals' life; it is required to build and maintain membranes. Moreover, it modulates membrane fluidity over the range of physiological temperatures [29]. In this work, decreased level of cholesterol may be due to systemic perturbations in metabolic processes of autoxidation, secondary oxidation to lipid peroxidation, and cholesterol-metabolizing enzyme oxidation; cause of cholesterol is susceptible to oxidation and easily forms oxygenated derivatives known as oxysterols [30]. The altered metabolite levels indicated significant metabolic changes in the energy metabolism, nucleic metabolism, lipid metabolism, and amino acid metabolism in H_2_S-poisoned rats and caused disorder.

The metabolic pathways of carbohydrate include anaerobic glycolysis of glucose, aerobic oxidation of phosphopentose, glycogen synthesis and breakdown, gluconeogenesis, and other hexose metabolisms [31]. The increased level of urine carbohydrates (d-mannose, arabinofuranose) indicated that chronic H_2_S poisoning induced the existence of carbohydrate metabolic disorders. Amino acids mainly play a role in the synthesis of proteins and polypeptides, which are mostly reabsorbed by the proximal renal tubule with little discharge in the urine. The increase of the concentration of glycine also suggested that the chronic H_2_S poisoning affects amino acid metabolism [32].

We have shown in this study the confounding influence of H_2_S poisoning in the urinary metabolite profiles in the H_2_S-poisoned and control rats. To our knowledge, this is the first report where metabolomics was applied in the detection of chronic H_2_S poisoning. However, it should be noted that the exact mechanisms leading to the observed metabolic changes should be further studied. In addition, a limitation of this study is that the rates of metabolism or cycling of a specific site in metabolic pathways that may be disrupted in the H_2_S poisoning progress were not provided, for the GC-MS measures used only suggest a static picture of metabolites measured.

## 4. Conclusion

In the present work we used GC-MS based metabonomic analysis to identify metabolic changes in urine extracts from H_2_S-poisoned and control rats. These biomarkers (alanine, d-ribose, L-aspartic acid, cholesterol, glycine, d-mannose, and arabinofuranose) were the additional evidence indicating that chronical H_2_S poisoning induced systemic perturbations of metabolic system in rats.

## Figures and Tables

**Table 1:** Summary of the changes in relative levels of metabolites in rat urine indicated by the PLS-DA loading plots and statistical analysis.

ID	Retention time	Metabolites compound	Mod versus Con
1	14.08	Alanine	↓^**^
2	15.70	d-Ribose	↓^**^
3	17.87	Tetradecanoic acid	↓^**^
4	18.31	Glycine	↑^**^
5	18.65	d-Mannose	↑^*^
6	18.87	L-Aspartic acid	↓^**^
7	18.09	Pentanedioic acid	↓^**^
8	20.54	Cholesterol	↓^**^
9	22.42	Acetate	↓^**^
10	22.62	Arabinofuranose	↑^**^
11	26.00	Oleic acid	↓^**^
12	26.08	Propanoic acid	↑^**^

Marks indicate the direction of the change, i.e., ↓ for decrease, ↑ for increase; ^*^ *P* < 0.05 and ^**^ *P* < 0.01, as indicated by the statistical analysis *t*-test.
